# Application of fluorescence correlation spectroscopy to investigate the dynamics of a ribosome-associated trigger factor in *Escherichia coli*

**DOI:** 10.3389/fmolb.2022.891128

**Published:** 2022-08-25

**Authors:** Tatsuya Niwa, Koki Nakazawa, Kensuke Hoshi, Hisashi Tadakuma, Koichi Ito, Hideki Taguchi

**Affiliations:** ^1^ School of Life Science and Technology, Tokyo Institute of Technology, Yokohama, Japan; ^2^ Cell Biology Center, Institute of Innovative Research, Tokyo Institute of Technology, Yokohama, Japan; ^3^ School of Life Science and Technology and Gene Editing Center, ShanghaiTech University, Shanghai, China; ^4^ Department of Computational Biology and Medical Sciences, Graduate School of Frontier Sciences, The University of Tokyo, Kashiwa, Japan

**Keywords:** molecular chaperone, trigger factor, ribosome, fluorescence correlation spectroscopy, co-translational folding, *in vitro* translation

## Abstract

Co-translational protein folding is one of the central topics in molecular biology. In *Escherichia coli*, trigger factor (TF) is a primary chaperone that facilitates co-translational folding by directly interacting with nascent polypeptide chains on translating ribosomes. In this study, we applied fluorescence correlation spectroscopy (FCS), which can analyze the diffusion properties of fluorescent molecules by measuring the fluctuations of the fluorescent intensity, to investigate the interaction between TF and a nascent chain on translating ribosomes both *in vitro* and *in vivo*. The FCS analysis with a reconstituted cell-free translation system revealed that the interaction of fluorescently labeled TF with a nascent chain depended on the emergence of the nascent chain from the ribosome exit tunnel, and this interaction was not inhibited by excess amounts of other chaperones. Furthermore, the translation-dependent interaction between GFP-fused TFs and nascent chains was also observed in living *E. coli* cells. The FCS-based approach established here could be an effective method to investigate the dynamics of other ribosome-associated chaperones besides TF.

## Introduction

In living organisms, proteins are essential biomolecules responsible for the functions to maintain life. For most proteins, proper folding dictated by the amino acid sequence is required to gain their functions (Anfinsen, 1973). In the cell, this process could start during translation. In both prokaryotes and eukaryotes, many chaperones interact with nascent polypeptides emerging from ribosomes to facilitate the proper folding of newly synthesized proteins (Balchin et al., 2016; Deuerling et al., 2019).

In *Escherichia coli*, the interactions between nascent chains and chaperones have been extensively investigated. The most prominent chaperone is assumed to be trigger factor (TF), which directly binds to the ribosome and an emerging nascent chain near the ribosomal exit tunnel to facilitate the folding of newly synthesized proteins by providing space for partial folding (Hoffmann et al., 2010; Balchin et al., 2016; Deuerling et al., 2019). Biochemical and structural studies showed that TF can weakly interact with the vacant ribosome, but this binding is largely strengthened by the existence of the nascent chains emerging from ribosomes (Kaiser et al., 2006; Rutkowska et al., 2008). In addition, this interaction is largely dependent on the properties of the nascent chains, such as lengths and sequences (Kaiser et al., 2006; Rutkowska et al., 2008; Lakshmipathy et al., 2010). These studies also demonstrated that the interactions are highly dynamic, with estimated dissociation half-times of 10∼35 s (Kaiser et al., 2006), 15∼53 s (Rutkowska et al., 2008), and ∼1.7 s (Bornemann et al., 2014). These length-dependent and dynamic interactions have also been investigated *in vivo* (Oh et al., 2011; Yang et al., 2016). In addition to TF, the DnaK/DnaJ chaperone could also interact with various nascent chains on ribosomes. Since the double deletion of DnaK/DnaJ and TF is lethal (Deuerling et al., 1999), DnaK/DnaJ is also considered to be a crucial chaperone for co-translational folding, in addition to TF.

Although the interactions between TF and nascent chains on ribosomes have been extensively studied, the analysis of the dynamic behavior of TF with another investigation method would be complementary and informative for understanding the nature of the interaction between TF and nascent chains. Accordingly, we applied fluorescence correlation spectroscopy (FCS), a technique that can observe the dynamic diffusion properties and the interactions between biomolecules, to study the interactions of TF with nascent chains emerging from the ribosome.

FCS is an optical method to analyze the diffusion of fluorescent molecules (Ries and Schwille, 2012; Diekmann and Hoischen, 2014). It measures the fluctuations of fluorescence intensity at fast time intervals in an extremely small detection volume, generated by confocal illumination. The fluctuations are analyzed by an autocorrelation function to extract the diffusion properties of fluorescent molecules of interest. Since these fluctuations are mainly derived from the Brownian motion of fluorescent molecules, the autocorrelation curve reflects the diffusion time of these molecules when the concentration of the fluorescent molecules is appropriate (typically between ∼10 nM and 1 µM). With ideal conditions and samples, an apparent diffusion coefficient (*D*) can be estimated by curve fitting. Another feature of FCS in biology is its noninvasive manner, which enables the application of FCS to living cells. Indeed, we previously conducted FCS measurements to analyze the dynamic behaviors of the yeast prion Sup35 (Kawai-Noma et al., 2006; Kawai-Noma et al., 2009; Kawai-Noma et al., 2010) and over 300 cytoplasmic GFP-fused proteins (Fukuda et al., 2019) in living *Saccharomyces cerevisiae* cells.

By using this technology, in this paper we sought to investigate the dynamic behavior of TF interacting with nascent chains during translation reactions both *in vitro* and *in vivo*. Our FCS analysis using a reconstituted cell-free translation system confirmed that the interaction between TF and nascent chains is dependent on the nascent chain length. In addition, the analysis in living *E. coli* cells demonstrated the applicability of FCS for investigating the translation-reaction-dependent interactions between TF and nascent chains.

## Materials and methods

### Plasmids and strains

The plasmids for TF expression and purification were derived from the pET29 vector harboring the *tig* sequence and a 6xHis-tag at the C-terminus, which was used in the previous study (Niwa et al., 2012). To introduce a fluorescent label, the arginine at the 14th residue was replaced with cysteine (R14C) (Kaiser et al., 2006) by a PCR-based site-directed mutation. The plasmid encoding the TF_FRK/AAA_-R14C mutant (Kramer et al., 2002) was also constructed by a PCR-based site-directed mutation. For *in vivo* expression in *E. coli*, the EGFP gene was inserted into the pACYC vector by using the *Nde*I/*Xho*I restriction enzyme sites. The TF (*tig*) -EGFP gene fusion was also inserted into the pACYC vector, with a GS linker sequence between the *tig* and EGFP genes. The mutation for TF_FRK/AAA_-EGFP was introduced by PCR-based site-directed mutagenesis. For *in vivo* observations of *E. coli*, the MG1655 strain was used. For *in vivo* observations of *S. cerevisiae*, the BY4741 strain harboring the YCplac-Gal1p-EGFP plasmid was used, as described previously (Fukuda et al., 2019).

### DNA/mRNA templates for *in vitro* translation

**FIGURE 1:**
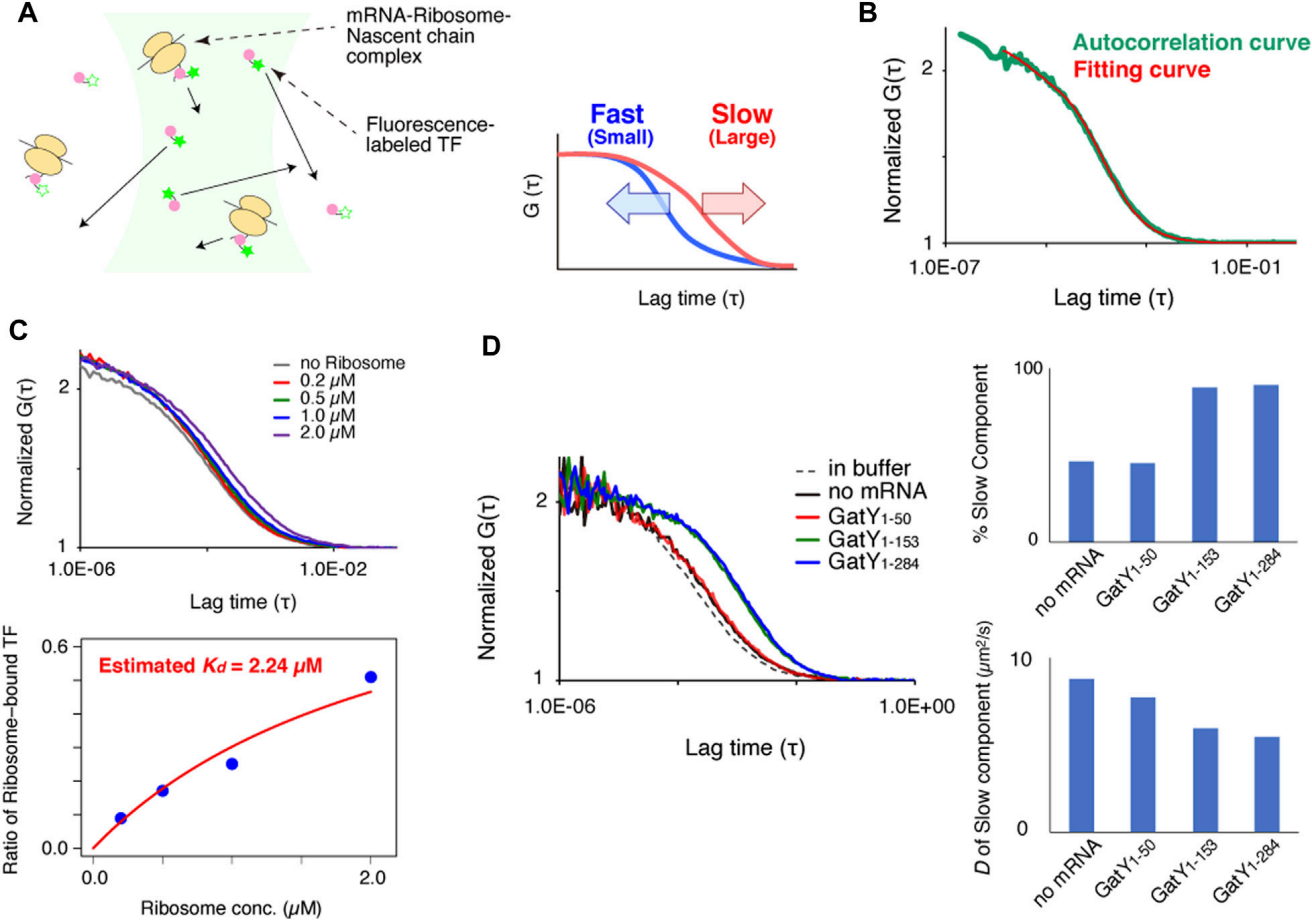
FCS measurement system for investigating interactions between trigger factor (TF) and the ribosome-nascent chain complex (RNC) *in vitro*. **(A)** Schematic illustration of the experimental procedure and the relationship between the autocorrelation curve obtained by FCS measurements and the diffusion time of the fluorescent molecules. **(B)** Normalized autocorrelation curve of Alexa488-labeled TF in buffer solution. Red lines indicates the fitting curve with a one-component model. The concentration of the Alexa488-labeled TF was 100 nM. **(C)** Investigation of the interaction between Alexa488-labeled TF and vacant ribosomes in buffer solution. The upper panel shows the normalized autocorrelation curves of Alexa488-labeled TF under each condition. The lower panel shows the ratios of ribosome-bound TF under each condition evaluated by a curve fitting with a two-component model. The dissociation constant (*K*_*d*_) was estimated by a fitting (red curve), shown in the method, as 2.24 µM. **(D)** Interactions between Alexa488-labeled TF and RNC after the translation reaction. The left panel shows normalized autocorrelation curves under each condition. The right panel represents the ratio of the slow component (upper panel) and diffusion coefficient (lower panel) evaluated by a curve fitting with a two-component model.

**FIGURE 2:**
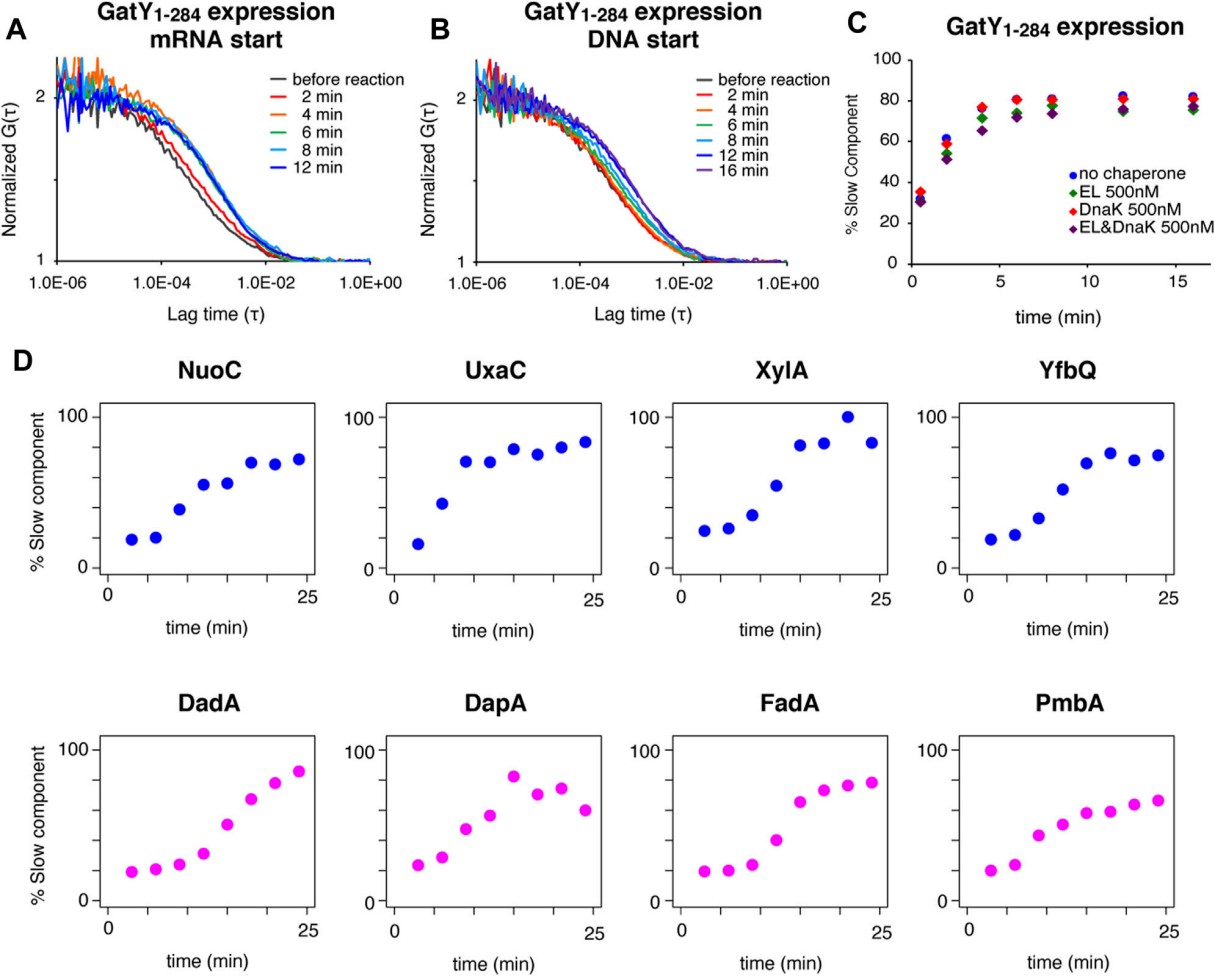
Real-time observation of interactions between TF and ribosome-nascent chain complex by FCS. **(A)** Autocorrelation curves at each reaction time of Alexa488-labeled TF during protein synthesis of *gatY*_1-284_ by the PURE system initiated by mRNA addition. **(B)** Autocorrelation curves at each reaction time of Alexa488-labeled TF during protein synthesis of *gatY*_1-284_ by the PURE system initiated by DNA addition. **(C)** Time-dependent changes in the populations of the slow diffusion component during protein synthesis of *gatY*_1-284_ by the PURE system. The population was obtained by curve fitting with a two-component model. The concentration of the Alexa488-labeled TF was 100 nM. When the excess amount of DnaK (500 nM) was added, DnaJ and GrpE were also added at 200 nM. **(D)** Time-dependent changes in the populations of the slow diffusion component during protein synthesis of each gene under the 0.2× EF-G concentration condition. The population was obtained by curve fitting with a two-component model. The upper (NuoC, UxaC, XylA, and YfbQ) and the lower (DadA, DapA, FadA, and PmbA) proteins have relatively hydrophobic and hydrophilic properties at the N-termini, respectively (see Supplementary Figure S2C for the hydropathy plots).

For use as a template in the PURE system for the FCS measurements in Figure 1 and Supplementary Figure S1 and TIRFM observations, the *gatY* gene was inserted into the pET21 vector with the external sequence including the HA-tag (MSYPYDVPDYAH) at the N-terminus. The genes for the time-course observation in Figure 2 and Supplementary Figure S2 (*gatY*_1-284_, *araA*_1-500_, *nuoC*_1-600_, *uxaC*_1-470_, *xylA*_1-440_, *yfbQ*_1-405_, *dadA*_1-432_, *dapA*_1-292_, *fadA*_1-387_, and *pmbA*_1-450_) were amplified by PCR from the plasmid used in the previous study (Fujiwara et al., 2010), harboring each gene located downstream of the *tac* promoter. Consequently, the T7 promoter sequence was attached at the 5′ UTR region by the primer. Truncated DNA fragments were prepared by PCR amplification with appropriate primers, as listed in Supplementary Table S1. The DNA fragment was then transcribed with a CUGA7 *in vitro* transcription kit (Nippon Gene, Japan). After the transcription, the mRNA was purified with an RNeasy MinElute Cleanup Kit (Qiagen, Germany). The purified mRNA was quantified by the absorbance at 260 nm. When DNA was used as a template instead of mRNA, the DNA fragment used for the transcription was employed.

### Protein purification and fluorescent labeling

The TF-R14C and TF_FRK/AAA_-R14C proteins were purified as described previously (Niwa et al., 2012). Briefly, the proteins were overexpressed in BL21 (DE3) cells. The expression was induced by 0.5 mM isopropyl-β-D-thiogalactopyranoside (IPTG) for ∼3 h at 37°C. The cells were disrupted by sonication, and the supernatant after high-speed centrifugation was collected for chromatography. The supernatant was applied to a HisTrap HP prepacked column (Cytiva, United States) for affinity purification. After elution by the imidazole gradient, the sample was further purified on a HiTrap Q HP column (Cytiva, United States). The labeling reactions of TF-R14C, TF_FRK/AAA_-R14C were conducted with a maleimide-fluorescent dye as the labeling agent. Before the reaction, the buffer was exchanged by passage through a gel filtration column (Micro Bio-Spin 6, Bio-Rad, United States) to remove the reducing agent. The protein sample was mixed with the labeling agent at a molar ratio of 1:1, and the mixture was incubated at room temperature for 2 h. After the reaction, the sample was applied to a gel filtration column to remove the unreacted labeling agent. As labeling agents, Alexa488 C_5_ Maleimide (Thermo Fisher Scientific, United States) and Alexa647 C_2_ Maleimide (Thermo Fisher Scientific, United States) were used. The labeling rates were 70∼100% for TF-R14C and TF_FRK/AAA_-R14C. Wildtype DnaK, DnaJ, GrpE, and GroEL for the competition assay (Figure 2C and Supplementary Figure S2A) were purified as described previously (Niwa et al., 2012).

### Fluorescence correlation spectroscopy measurements *in vitro*

The ribosome-nascent chain complex (RNC) for FCS measurements *in vitro* were prepared with a PURE*frex* ver.1 cell-free protein synthesis kit (GeneFrontier Corporation, Japan). For endpoint assays (Figure 1 and Supplementary Figure S1), ∼2 µg of truncated mRNA and 100 nM of fluorescently-labeled chaperone were added to the PURE*frex* solution, which was then incubated for 5∼15 min at 37°C. After the reaction, the solution was transferred to an 8-well chamber slide (Nunc 155411, Thermo Fisher Scientific, United States) for FCS measurements. For real-time observations (Figure 2 and Supplementary Figure S2), 100 nM of fluorescence-labeled TF was added to the PURE*frex* solution and incubated at 37°C for 10 min, as a pre-incubation step. The solution was then transferred to a chamber slide and ∼2 µg of truncated mRNA was added to initiate the reaction. For the DNA-start observation (Figure 2B), ∼40 ng of DNA was added instead of mRNA. For the observations under the lower EF-G concentration, the customized PURE*frex* kit was used. For forming the initiation complex, initiation factors 1, 2, and 3, methionyl-tRNA synthetase, methionyl-tRNA formyltransferase, and ribosomes were mixed with mRNA (equimolar amounts to ribosomes). Then, the PURE reaction buffer was mixed with 100 nM of fluorescently-labeled TF and incubated at 37°C for 10 min. At the same time, the elongation factor mixture (EF-Tu, Ts, and G, 19 aminoacyl-tRNA synthetases, and an energy regeneration system) in the PURE reaction buffer were also incubated at 37°C for over 5 min. Then, the two solutions were mixed in the slide chamber to initiate the reaction. The final concentration of ribosomes in the reaction mixture was 500 nM. To delay the translation elongation reaction, we used the 0.2× EF-G concentration of the standard kit. For observations of fluorescently-labeled TF and ribosomes in buffer solution, 100 nM of fluorescently-labeled TF and represented concentration of ribosomes were diluted in HKM buffer (25 mM Hepes-KOH (pH 7.4), 100 mM KCl, 5 mM MgCl_2_).

The FCS measurements were performed with an LSM780 confocal laser scanning microscope system, equipped with a GaAsP detector (Carl Zeiss, Germany). The objective lens was a C-Apochromat ×40/1.2 water immersion lens. Before the measurement, the correction collar of the objective lens was adjusted to be optimal for each chamber slide. The excitation laser was a 488 nm argon laser. The FCS measurements were acquired five or ten times, for five or ten seconds each. The measurements with unusually large fluctuating signals derived from large aggregates were excluded. Before the sample measurement, a 10^−7^ M rhodamine 6G solution was measured to obtain the structure parameter every measurement day. All data acquisition and curve fitting were performed with the ZEN software (Carl Zeiss, Germany). All the autocorrelation curves and the fitting curves were listed in the Appendix information provided as a Supplementary Material. The dissociation constant (*K*_*d*_) between TF and the ribosome (Rbs) was estimated by the following formula:

$$K_{d}=\frac{\left[TF\right]\cdot \left[Rbs\right]}{\left[TF-\mathrm{Rbs}\right]}$$ (1)

The input ribosome concentration (*x*) and the ratio of the ribosome-bound TF (*y*) can be represented as:

x=[Rbs]+[TF-Rbs] (2)

$$y=\frac{\left[TF-\mathrm{Rbs}\right]}{{\left[TF\right]}_{0}}$$ (3)

where [TF]_0_ denotes the initial concentration of TF. Then, organizing this equation as a quadratic equation for y:

$$y^{2}-\left(1+\frac{x}{{\left[TF\right]}_{0}}+\frac{K_{d}}{{\left[TF\right]}_{0}}\right)\cdot y+\frac{x}{{\left[TF\right]}_{0}}=0$$ (4)

By applying the formula for solving quadratic equation (since y must be less than 1, the solution is uniquely determined):

$$y=\frac{1}{2}\cdot \left\{\frac{x}{{\left[TF\right]}_{0}}+\frac{K_{d}}{{\left[TF\right]}_{0}}+1-\sqrt{{\left(\frac{x}{{\left[TF\right]}_{0}}\right)}^{2}+\frac{2}{{\left[TF\right]}_{0}}\cdot \left(\frac{K_{d}}{{\left[TF\right]}_{0}}-1\right)\cdot x+{\left(\frac{K_{d}}{{\left[TF\right]}_{0}}+1\right)}^{2}}\right\}$$ (5)

This formula was used for the fitting by R.app (for macOS, ver. 4.1.2) with the nls function to estimate the value of *K*_*d*_ ([TF]_0_ = 0.1 (µM) from the experimental condition).

### Single molecule fluorescence observation by total internal reflection fluorescence microscopy

Single molecule fluorescence observations by TIRFM were performed as described previously, with several modifications (Taguchi et al., 2001; Ueno et al., 2004; Zhou et al., 2011). The RNCs were prepared as described above. For visualization, a TMR-labeled Halo-S2 ribosome (Zhou et al., 2011) was used, instead of a normal 70S ribosome, at a 50 nM concentration. The concentrations of the Alexa647-labeled TF-R14C and TF_FRK/AAA_-R14C were 50 nM.

For the TIRFM observations, a flow chamber consisting of a polyethylene glycol (PEG)-coated quartz glass slide and cover glass was used. PEG-coated quartz glass slides were prepared as previously described (Yokota et al., 2009). A 1/2000 amount of biotinylated PEG against PEG was mixed in the PEG-coating solution. To immobilize RNCs, 0.33 mg/ml neutravidin solution was poured into the flow chamber, which was then washed with HKM buffer. The sample solution was then poured into the flow chamber. For immobilization, anti-HA biotin (3F10, Roche, Switzerland) was added to the sample at 2.5 ng/µl before it was poured into the flow chamber. To prevent photobleaching, an O_2_ scavenger system (0.5 U/ml glucose oxidase, 0.5 U/ml catalase, 0.045 mg/ml glucose and 0.1 mM DTT) was also added to the sample.

The fluorescence images were obtained with a total internal reflection fluorescence microscope system based on an inverted microscope (IX70; Olympus, Japan). The lasers for the stimulation of fluorescent molecules were a Sapphire 532 LP (Coherent, United States) for TMR and a 633 nm He-Ne laser (Coherent, United States). Fluorescence images were separated into two channels by using a Dual-View imaging system (Optical Insights, United States) to observe the fluorescence signals derived from TMR and Alexa647 separately, at the same time. The separated side-by-side images were acquired with an electron-multiplying charge coupled device camera (iXon X3, DU897, Andor Technology, United Kingdom) controlled by the Soris software (Andor Technology, United Kingdom). Images were acquired at a frame rate of 10 frames/sec for 150 s, and were analyzed with the ImageJ software (https://imagej.nih.gov/ij/). Firstly, the image was divided into the TMR-ribosome channel and the Alexa647-chaperone channel. The TMR-ribosome images were averaged into one picture (Z project, Average intensity). The Alexa647-chaperone images were smoothed by the rolling average method (with the Running ZProjector plugin (https://valelab4.ucsf.edu/∼nstuurman/IJplugins/Running_ZProjector.html), size = 10, Average intensity). To set the positions of the TMR-ribosomes, the circular regions of interest (ROIs) were generated by the Time Series Analyzer plugin (https://imagej.nih.gov/ij/plugins/time-series.html). The diameter of the ROIs was set at five pixels. The time course intensity of the Alexa647-chaperone on each ROI was obtained by the Time Series Analyzer. The time course signal was then further analyzed to calculate the duration time by our in-house macro program (Taguchi et al., 2001; Ueno et al., 2004). The fitting analysis was performed by R.app (for macOS, ver. 4.1.2) with the nls function.

### Fluorescence correlation spectroscopy measurements *in vivo*

*E. coli* cells were cultured in TB medium (1% tryptone and 0.5% NaCl), and protein expression was induced by 0.1 mM IPTG for 4 hours. Afterwards, 2 µg/ml of piperacillin was added to the culture, 1 hour before the observation. For the translation-inhibited conditions, an antibiotic cocktail consisting of 50 µg/ml kasugamycin, 100 µg/ml erythromycin, and 10 µg/ml puromycin was added, 20 min before the observation. About 2 µL of the culture was placed onto the chamber slide, and then a piece of 1% agarose gel prepared with TB medium was placed on the solution to fix the cells. The FCS measurements were acquired with an LSM780 confocal laser scanning microscope system, as described above. The data were acquired seven times, for 5 seconds in each measurement. The data that showed strong photobleaching and high fluctuations were removed before the fitting analysis.

## Results and discussion

### Fluorescence correlation spectroscopy analysis of chaperones in buffer solution

Our strategy of using FCS to evaluate the interactions between chaperones and nascent chains emerging from ribosomes is schematically depicted in Figure 1A. When chaperones are labeled with fluorescent dyes or fused with fluorescent proteins, the free chaperones diffuse quickly, while those bound to the ribosome-nascent chain complex (RNC) diffuse slowly because of their larger sizes. As shown in the right panel of Figure 1A, the fluorescence autocorrelation curve of fast-moving molecules decreases at a shorter lag time, whereas that of slow-moving molecules shifts to the right because its autocorrelation is maintained with a longer lag time. If the size of the fluorescent molecules is nearly homogeneous, then the diffusion time can be estimated by curve fitting of the autocorrelation curve with a one-component model. However, if two or three components with different diffusion times exist in the solution, then the diffusion time and the population of each component can only be estimated by fitting when the difference between the diffusion times is large enough. If more components with different diffusion times exist, then only qualitative information about their diffusion times can be obtained from the shapes of the autocorrelation curves.

Before the observations of the interaction between TF and nascent chains on ribosomes, 100 nM of fluorescently-labeled TF was measured in a buffer solution. Since the autocorrelation curve was fitted well with the equation of a one-component model, TF was considered to exist in a uniform state in the buffer solution at this concentration (Figure 1B). By comparing the obtained diffusion coefficients with that of the rhodamine-6G standard and its molecular weight, a hypothetical molecular weight can be estimated. The estimated molecular weight of TF was in good agreement with the actual molecular weight from the amino acid sequence (*D* = 57.6 ± 0.5 µm^2^ s^−1^ and estimated MW = 55.0 ± 1.6 kDa, *n* = 5, SEM).

Then, we investigated the interaction between TF and vacant ribosomes in the buffer solution. The autocorrelation curve of fluorescently-labeled TF shifted toward the larger area as the concentration of ribosomes increased (Figure 1C). The dissociation constant (*K*_*d*_) obtained by the curve fitting with the values of the ratio of TF bound to ribosomes, evaluated by a two-component fitting of the autocorrelation curve, was 2.24 µM, which is consistent with the previous reports (Kaiser et al., 2006; Hoffmann et al., 2010) (Figure 1C).

### Fluorescence correlation spectroscopy analysis of the TF-RNC interaction under reconstituted cell-free translation conditions

To investigate the interaction between nascent chains on ribosomes and TF, a truncated mRNA that lacked a stop codon at the C-terminus was translated by the PURE system, a reconstituted cell-free translation system (Shimizu et al., 2001). As the model substrate, GatY (D-tagatose-1,6-bisphosphate aldolase subunit) from *E. coli* was used because it has a strong tendency to form aggregates when translated by the chaperone-free PURE system (Niwa et al., 2009) and its folding obligately depends on the GroEL/GroES chaperonin *in vivo* (Fujiwara et al., 2010). To evaluate the length-dependency of the nascent chains, three truncated mRNAs with different lengths were translated by the PURE system supplemented with fluorescently-labeled TF. The autocorrelation curve after the translation of *gatY*_1-50_ was almost the same as that of a control experiment without mRNA, suggesting that the fragment of GatY (GatY_1-50_) does not contribute to the interaction with TF on ribosomes (Figure 1D, left panel). This is probably because the length of the nascent chain is too short to reach the outside of the ribosome exit tunnel. In contrast, the autocorrelation curve of nascent chains from *gatY*_1-153_ and full-length *gatY* gene (*gatY*_1-284_) shifted toward larger, indicating the increase of molecules with slower diffusion (Figure 1D, left panel). Note that the translated nascent chains should remain on ribosomes even in the translation of the full-length *gatY* since the truncated mRNAs, which do not have stop codon at the 3′-end, were used to prevent translation termination reaction in this experimental setup. We then investigated the ratio of the slower component and its diffusion coefficient by a curve fitting with the two-component model in which the diffusion time of the fast component was fixed to the value of freely diffusing TF. The result showed that ∼90% of TF diffused as the slow component, although the molecular weight estimated from the diffusion coefficient was more than ten times larger than that of a single ribosome (Figure 1D, right panel). The extremely slow diffusion may be due to the flexible structure of mRNAs on ribosomes.

The interaction between TF and the nascent chain that emerged from the ribosome was confirmed as follows. First, the addition of puromycin, a reagent that dissociates nascent chains from the ribosomes, shifted the autocorrelation curve to the left after the translation of *gatY*_1-284_ (Supplementary Figure S1A), suggesting that the change in TF diffusion is derived from the interaction with the nascent chains emerging from the ribosomes. Second, the TF interaction with RNC was almost abolished when a TF mutant (TF_FRK/AAA_), which cannot interact with the ribosome (Kramer et al., 2002), was used instead of wild-type TF (Supplementary Figure S1B). Collectively, we concluded that the interaction between TF and nascent chains can be investigated by FCS measurements *in vitro*.

The length-dependent interaction between TF and nascent chains was also confirmed by single-molecule observations, using total internal reflection fluorescence microscopy (TIRFM). In this analysis, RNCs prepared by the PURE system with truncated mRNA were fixed on the surface of a glass slide via a monoclonal antibody and N-terminal epitope tags (Supplementary Figure S1D). Tetramethylrhodamine (TMR)-labeled ribosomes were used to determine the position of the RNC. The dynamic binding and dissociation were observed in a length-dependent manner by this TIRFM observation (Supplementary Figure S1E). The half-life of the residence time of the GatY_1-153_ nascent chain estimated by the fitting showed that the fast component was dominant (∼2.7 s, 92%) (Supplementary Figure S1F). This result supports the fast dissociation (∼1.7 s) reported previously (Bornemann et al., 2014). Note that an increase in the slow fraction (9.8 s, 37%) was observed with the GatY_1-284_ nascent chain (Supplementary Figure S1F), confirming that the interaction of TF with nascent chains highly depends on the state of the nascent chain (Kaiser et al., 2006; Rutkowska et al., 2008; Lakshmipathy et al., 2010).

### Features of the TF-RNC interaction revealed by the *in vitro* fluorescence correlation spectroscopy analyses

Using the established *in vitro* FCS analysis system, we examined when TF interacts with the growing nascent chains during the translation reaction. The translation reaction was started by adding truncated mRNA to the slide chamber. The FCS analysis showed that the interaction became apparent after two minutes, and was almost saturated at four to six minutes (Figure 2A). This result demonstrated that the translation in the PURE system starts and proceeds rapidly, and a sufficient length of the nascent chain for the interaction with TF emerged within a few minutes. When DNA was used as the translation template instead of mRNA, the increase of the interaction was significantly delayed (Figure 2B). This result suggests that the transcription step is dominant during the first few minutes in the translation by the PURE system, in a transcription-translation coupled manner.

We next investigated whether the TF-RNC interaction is competitively affected by other chaperones. In this analysis, a time-course investigation of fluorescently-labeled TF was performed in the presence of a five-fold amount of non-labeled GroEL and/or DnaK chaperone system. To facilitate the comparison, all time-course data were fitted as a two-component model in which both the diffusion times of the fast and slow components were fixed. The population of the slow component, which is derived from the interaction between TF and RNC, increased with the reaction time (Figure 2C). Importantly, this increase in the population of the slow component did not change, even in the presence of excess amounts of GroEL and/or DnaK (Figure 2C). The same tendency was observed when the model protein was changed from GatY to AraA (L-arabinose isomerase), another aggregation-prone protein and an obligate GroE-dependent substrate in *E. coli* (Supplementary Figure S2A). These results suggest that the interaction between TF and nascent chains is not affected by the presence of excess amounts of other chaperones, confirming that TF is the primary chaperone for nascent chains emerging from ribosomes, even for obligate GroE-dependent substrates.

Since the hydrophobic property is assumed to be one of the main factors in the TF-ribosome dynamics (Kaiser et al., 2006; Rutkowska et al., 2008), we then investigated whether the translation of proteins with different hydrophobicities affects the kinetics of the interaction between TF and the translating ribosome. Since the interaction started too fast with the normal PURE system, we used a customized PURE system containing a lower (0.2×) concentration of elongation factor G (EF-G) to uniformly delay the translation elongation. In this analysis, to make the translation elongation as uniform as possible, the factors necessary for forming the initiation complex were mixed and incubated before the reaction. After that, the factors necessary for translation elongation were added to start the reaction. As expected, the increase of the slower component of TF was largely delayed under this condition (Supplementary Figure S2B). Four proteins (NuoC, UxaC, XylA, and YfbQ) with lower hydrophobicity and four proteins (DadA, DapA, FadA, and PmbAs) with higher hydrophobicity at their N- terminal region were chosen among GroE class IV substrates, which have a strong tendency to form aggregates without chaperones (Supplementary Figure S2C). The observation for the eight substrate proteins showed that the time to initiate the interaction with TF reproducibly varied among substrates regardless of their N-terminal hydrophobicity (Figure 2D; Supplementary Figure S2D). These results suggest that local hydrophobicity at the N-terminal region is not a dominant factor in the strong interaction with TF on ribosomes, suggesting that other properties would contribute to the interaction. Since translation factors and tRNAs are abundant in the PURE system and only EF-G concentration was reduced for the delay of the elongation reaction, the variation in translation elongation speed among substrate proteins should be minimized. However, we cannot completely rule out the possibility that some unexpected specific interaction between nascent chains and the inside of the ribosome tunnel may exist.

### *In vivo* fluorescence correlation spectroscopy analysis of the diffusion behavior of trigger factor

**FIGURE 3:**
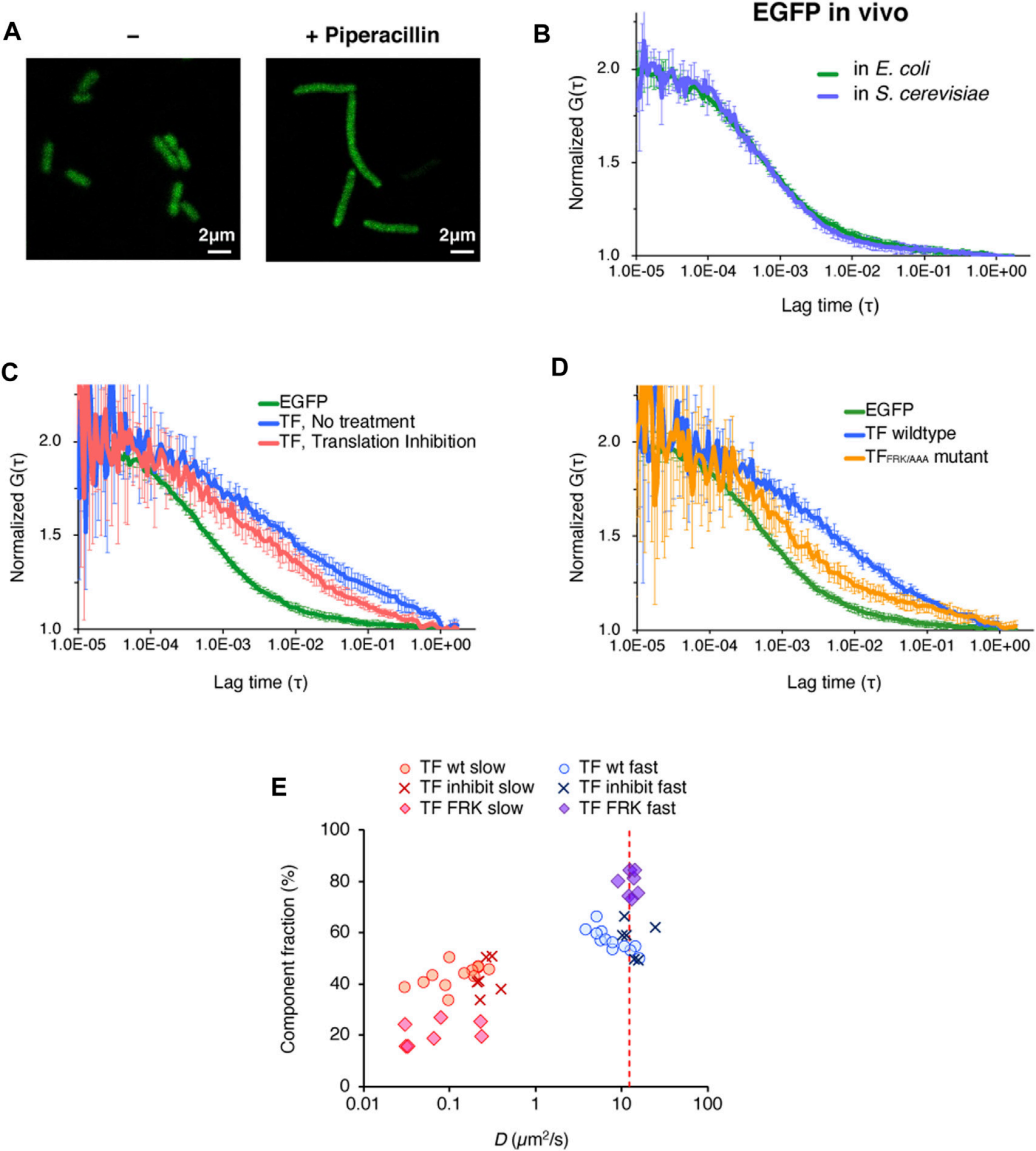
*In vivo* observations of the behavior of TF-EGFP by FCS. **(A)** Fluorescence image of *E. coli* cells before and after the piperacillin treatment. This treatment caused the cells to elongate into a filamentous shape. Scale bar = 2 µm. **(B)** Averaged autocorrelation curves of EGFP observed in *E. coli* cells and *S. cerevisiae* cells. The number of observed cells was ten for *E. coli* cells and five for *S. cerevisiae* cells. Error bars represent standard deviations. **(C)** Averaged autocorrelation curve of TF-EGFP observed in *E. coli* cells. For the comparison, the autocorrelation curve of EGFP is shown again (the same as depicted in Figure 3B). Translation inhibition was accomplished by the treatment with a mixture of antibiotics (50 µg/ml kasugamycin, 100 µg/ml erythromycin, and 10 µg/ml puromycin). The number of the observed cells was five for the no treatment condition and six for the translation inhibition condition. Error bars represent standard deviations. **(D)** Averaged autocorrelation curves of EGFP-fused TF and a TF mutant (TF_FRK/AAA_) observed in *E. coli* cells. For the comparison, the autocorrelation curve of EGFP is shown again (the same as depicted in Figure 3B). The number of observed cells was five for the no treatment condition and seven for the translation inhibition condition. Error bars represent standard deviations. **(E)** Distribution of the diffusion coefficients and the populations of both the fast and slow components under each condition, obtained by two-component fitting. The red dashed line represents the hypothesized diffusion coefficient of TF-EGFP, calculated by its molecular weight (75 kDa), and the diffusion coefficient of EGFP in *E. coli* cells obtained in this study.

Since the FCS measurement can also be applied to living cells, we next investigated the chaperone action on nascent chains in living *E. coli* cells. However, since the *E. coli* cell is quite small, photobleaching during the measurement is inevitable and thus accurate measurements are nearly impossible. To overcome this problem, we decided to elongate the cells by treating them with piperacillin, an antibiotic that inhibits the biosynthesis of bacterial cell walls (Pogliano et al., 1997) (Figure 3A).

We first investigated the diffusion of enhanced GFP (EGFP) expressed in the filamentous *E. coli* cells. We acquired sufficient amounts of the fluorescence fluctuation signals in the FCS measurement, and the autocorrelation curve of EGFP was quite similar to that measured in *S. cerevisiae* cells (Figure 3B). The diffusion coefficient of the fast component, calculated by two-component curve fitting, was in close agreement with that in *S. cerevisiae* cells (Table 1 and Supplementary Figure S3A), and consistent with the value reported in a previous *S. cerevisiae* study (Fukuda et al., 2019).

**TABLE 1:** Apparent diffusion coefficients (*D*) of GFP and GFP-fused trigger factor (TF) *in vivo*.

Protein	Organism/Condition	*D*_fast_ (µm^2^s^−1^) (%)	*D*_slow_ (µm^2^s^−1^) (%)
EGFP	*E. coli*	17.2 ± 1.9 (86 ± 4)	0.60 ± 0.49 (14 ± 4)
	*S. cerevisiae* (This study)	16.3 ± 2.4 (92 ± 3)	1.03 ± 1.28 (8 ± 4)
	*S. cerevisiae* Fukuda T. et al., 2019	17.7 ± 0.6 (92 ± 1)	0.3 ± 0.1 (8 ± 1)
TF-EGFP	Wildtype	8.6 ± 4.1 (57 ± 4)	0.14 ± 0.08 (43 ± 4)
	Translation inhibition	14.5 ± 5.4 (58 ± 7)	0.27 ± 0.07 (42 ± 4)
	FRK/AAA mutant	12.6 ± 2.4 (79 ± 4)	0.12 ± 0.10 (21 ± 4)

Data are presented as the means ± standard deviation of the values obtained from each cell. The numbers of analyzed cells (*N*) were as follows: *N* = 10 for EGFP in *E. coli* and TF-EGFP, *N* = 5 for EGFP in *S. cerevisiae*, *N* = 6 for TF-EGFP under translation inhibition conditions, and *N* = 7 for the FRK/AAA mutant of TF-EGFP.

We then examined the behavior of TF in the *E. coli* cell. As shown in Figure 3C, the diffusion time of EGFP-fused TF at the C-terminus (TF-EGFP) was much larger than that of EGFP, even if we subtracted the difference of their molecular weights as monomers. When protein synthesis in the cell was impeded by a cocktail of antibiotics that inhibit translation, this slower diffusion property was partially eliminated (Figure 3C). The diffusion time showed a further decrease when the TF mutant (EGFP-fused TF_FRK/AAA_) was used instead of wildtype TF (Figure 3D). These results indicate that at least some population of TF in the cell interacts with nascent chains emerging from ribosomes in a translation-coupled manner.

Although the values may not be accurate since the autocorrelation curve of TF-EGFP highly fluctuated compared to that of EGFP *in vivo*, we tried to perform a semi-quantitative analysis by curve fitting. The estimated diffusion coefficient of the fast component in TF-EGFP was lower than the calculated diffusion coefficients estimated by the actual molecular weight of TF-EGFP (Figure 3E; Table 1 and Supplementary Figure S3B). In addition, the diffusion coefficients of both the fast and slow components were widely dispersed in TF-EGFP (Figure 3E and Table 1). This dispersion was presumably due to the heterogeneous diffusion states of TF originating from the various sizes of its complexes in the cell. The comparison between the conditions showed that the inhibition of protein synthesis led to increases in the diffusion coefficients of both the fast and slow components (*p* = 0.046 for the fast component and *p* = 0.00046 for the slow component by Wilcoxon’s rank sum test, Figure 3E and Table 1). Meanwhile, the loss of the binding activity to ribosomes in the TF_FRK/AAA_ mutant induced an increase in the fast population, but the slow diffusion species still existed although in smaller proportions (*p* = 0.0073 for the fast component and *p* = 0.35 for the slow component by Wilcoxon’s rank sum test, Figure 3E and Table 1). Note that TF-EGFP may form the dimer state with endogenous TF *in vivo* (*K*_*d*_ = 1∼2 µM) (Kaiser et al., 2006; Hoffmann et al., 2010), since the concentration of endogenous TF is thought to be high (∼50 µM) (Lakshmipathy et al., 2010). However, FCS is not sensitive to distinguish such a small size change since the diffusion coefficient is proportional to the cubic root of the molecular size. Thus, our FCS observation cannot differentiate between monomer and dimer states, and the fast component in this analysis is thought to include both monomer and dimer states. The previous single-molecule tracking (SMT) study suggested the existence of three diffusion states of TF *in vivo*, with estimated intrinsic diffusion coefficients of ∼11, 0.2, and 0.02 μm^2^ s^−1^ (Yang et al., 2016). Our FCS observations are partially consistent with the previous results (*D*_fast_ = 8.6∼14.5 μm^2^ s^−1^ and *D*_slow_ = 0.1∼0.3 μm^2^ s^−1^, see Table 1), although FCS could not separate the two slower components well and the populations of the fast fraction did not match well (20 ± 2% by SMT and ∼60% by FCS, see Table 1). This inconsistency is presumably due to differences in experimental methods or cellular states. However, the diffusion state of TF is thought to be more complex, so further investigations are needed. Thus, although the quantitative estimation is still somewhat ambiguous, FCS measurements are effective to investigate the interactions between TF and the ribosome-nascent chain complex both *in vitro* and *in vivo*.

## Conclusion and perspective

In this analysis, we demonstrated the applicability of FCS measurements for investigations of TF and ribosome-nascent chain complexes. The advantage of FCS measurement is that it is basically highly sensitive and relatively easy to measure if the equipment is available. In addition to the *in vitro* analysis, FCS can be applied to a living cell in a non-invasive manner. It can also chase the time-course changes in reactions at a minute level, as shown in our observations. In contrast, FCS may not be suitable for the investigation of small changes in diffusion rate or precise investigation with many components. Due to these limitations, precise quantification remains difficult in some cases, but FCS can investigate the overall changes in the diffusion behaviors of proteins or other biomolecules in various situations. Our results revealed that FCS measurements can be applied to complex reaction systems, such as translation *in vitro* and in small living cells like *E. coli* to some extent. Although FCS still has some limitations, this method has great potential for analyses of the various dynamic interactions and behaviors of biomolecules in real time, both *in vitro* and *in vivo*.

## Data Availability

The datasets presented in this study can be found in online repositories. The names of the repository/repositories and accession number(s) can be found in the article/Supplementary Material.

## Abbreviations

FCS, fluorescence correlation spectroscopy; RNC, ribosome-nascent chain complex; TF, Trigger Factor; TIRFM, total internal reflection fluorescence microscopy; TMR, tetramethylrhodamine.
