# Supplementary material for: Application of fluorescence correlation spectroscopy to investigate the dynamics of a ribosome-associated trigger factor in Escherichia coli
Source: Front Mol Biosci. 2022 Aug 25;9:891128. doi: 10.3389/fmolb.2022.891128 (PMC9452904; doi:10.3389/fmolb.2022.891128)
Supplement: Supplementary file 1 [file DataSheet2.pdf]

Appendix of

Application of fluorescence correlation spectroscopy to investigate the dynamics of a ribosome-associated trigger factor in *Escherichia coli.*

Tatsuya Niwa[1,2], Koki Nakazawa[1], Kensuke Hoshi[1], Hisashi Tadakuma[3], Koichi Ito[4], and Hideki Taguchi[1,2,*]

[1] School of Life Science and Technology, Tokyo Institute of Technology, Yokohama 226-8503, Japan
[2] Cell Biology Center, Institute of Innovative Research, Tokyo Institute of Technology, Yokohama 226-8503, Japan
[3] School of Life Science and Technology & Gene Editing Center, ShanghaiTech University, Shanghai 201210, China
[4] Department of Computational Biology & Medical Sciences, Graduate School of Frontier Sciences, The University of Tokyo, Kashiwa, Chiba 277-8562, Japan

*Correspondence to: taguchi@bio.titech.ac.jp

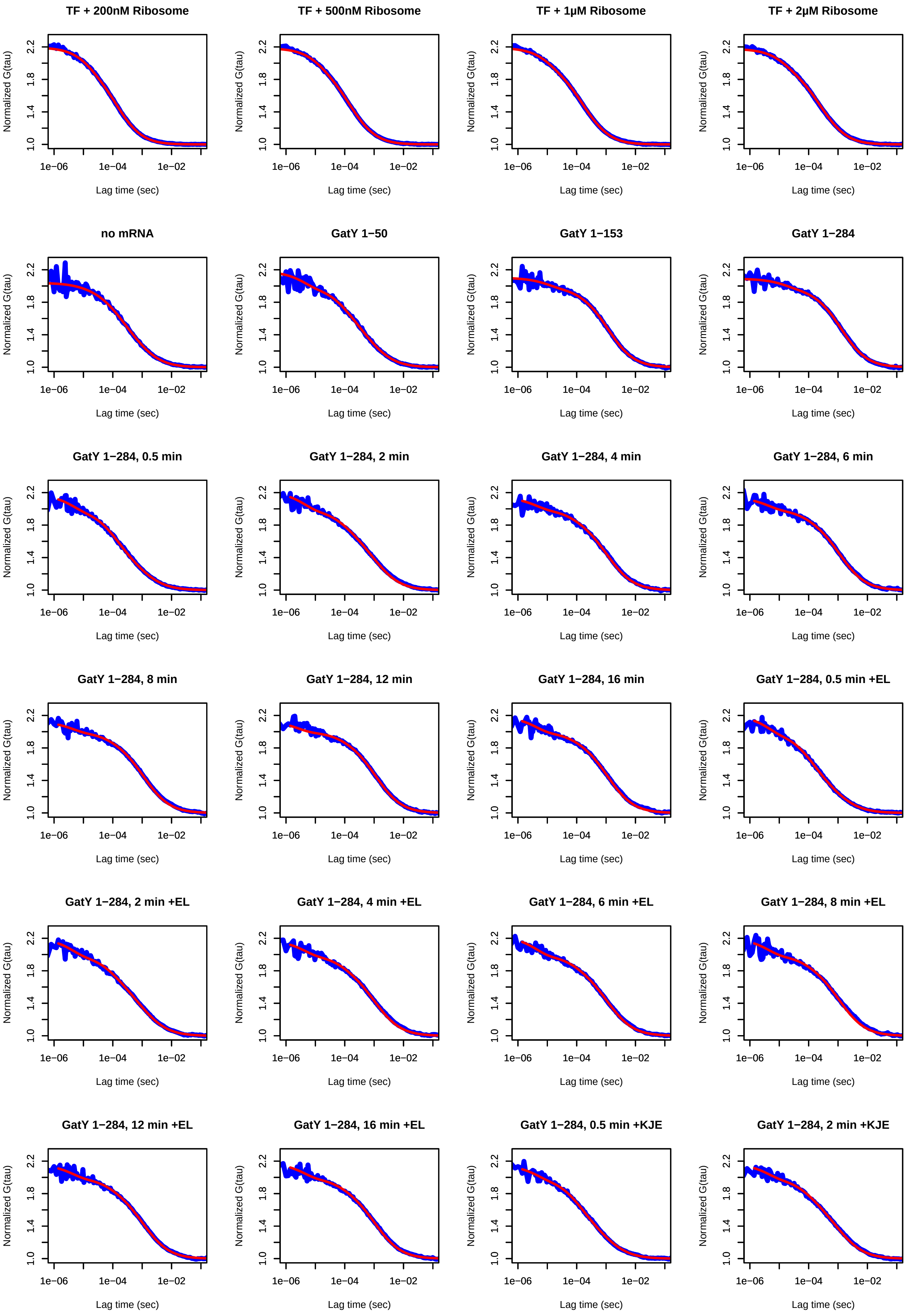
TF + 200nM Ribosome
TF + 500nM Ribosome
TF + 1μM Ribosome
TF + 2μM Ribosome
no mRNA
GatY 1−50
GatY 1−153
GatY 1−284
GatY 1−284, 0.5 min
GatY 1−284, 2 min
GatY 1−284, 4 min
GatY 1−284, 6 min
GatY 1−284, 8 min
GatY 1−284, 12 min
GatY 1−284, 16 min
GatY 1−284, 0.5 min +EL
GatY 1−284, 2 min +EL
GatY 1−284, 4 min +EL
GatY 1−284, 6 min +EL
GatY 1−284, 8 min +EL
GatY 1−284, 12 min +EL
GatY 1−284, 16 min +EL
GatY 1−284, 0.5 min +KJE
GatY 1−284, 2 min +KJE
Normalized G(tau)
1.0
1.4
1.8
2.2
Lag time (sec)
1e−06
1e−04
1e−02

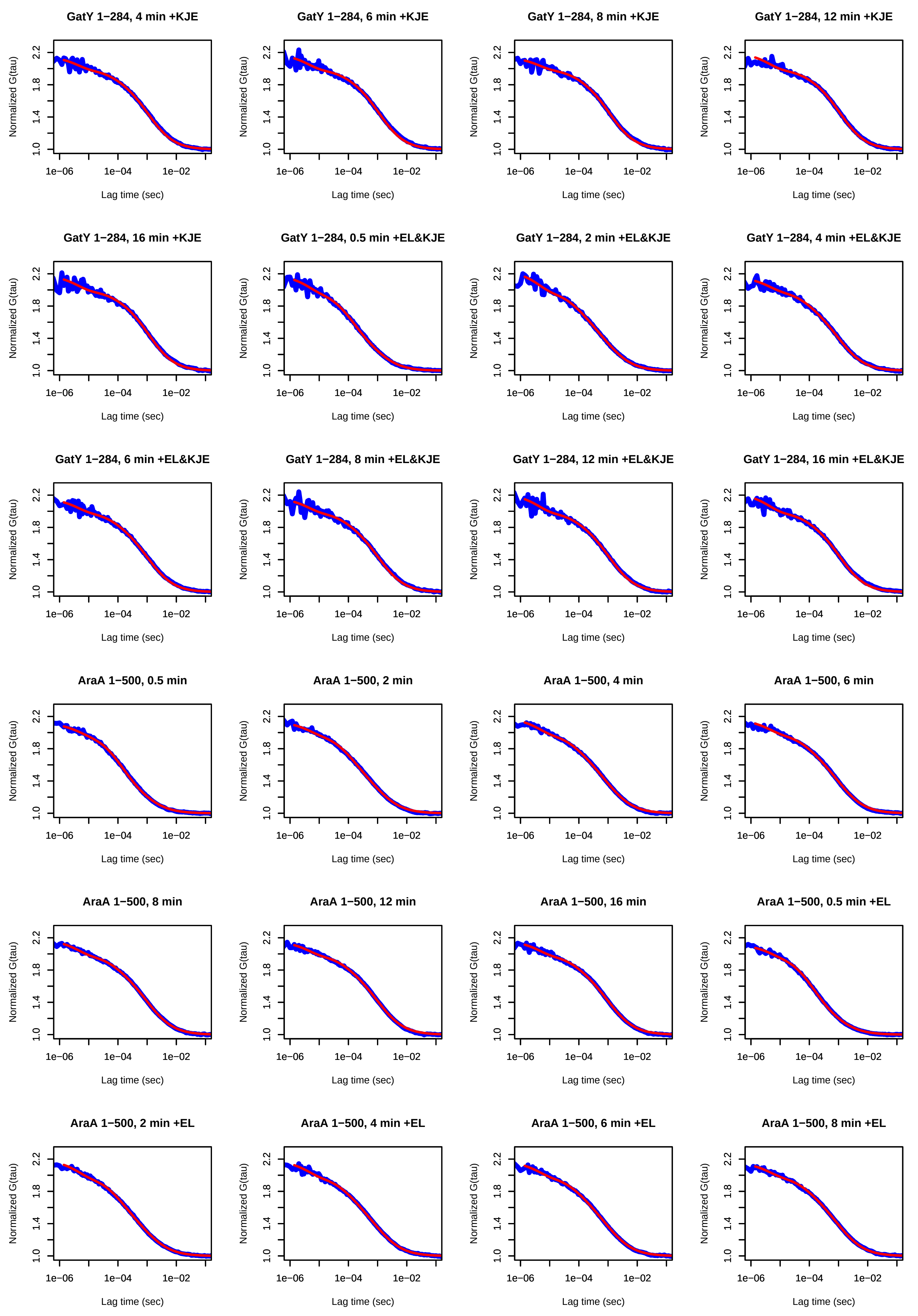
GatY 1−284, 4 min +KJE
GatY 1−284, 6 min +KJE
GatY 1−284, 8 min +KJE
GatY 1−284, 12 min +KJE
GatY 1−284, 16 min +KJE
GatY 1−284, 0.5 min +EL&KJE
GatY 1−284, 2 min +EL&KJE
GatY 1−284, 4 min +EL&KJE
GatY 1−284, 6 min +EL&KJE
GatY 1−284, 8 min +EL&KJE
GatY 1−284, 12 min +EL&KJE
GatY 1−284, 16 min +EL&KJE
AraA 1−500, 0.5 min
AraA 1−500, 2 min
AraA 1−500, 4 min
AraA 1−500, 6 min
AraA 1−500, 8 min
AraA 1−500, 12 min
AraA 1−500, 16 min
AraA 1−500, 0.5 min +EL
AraA 1−500, 2 min +EL
AraA 1−500, 4 min +EL
AraA 1−500, 6 min +EL
AraA 1−500, 8 min +EL
Normalized G(tau)
1.0
1.4
1.8
2.2
1e−06
1e−04
1e−02
Lag time (sec)

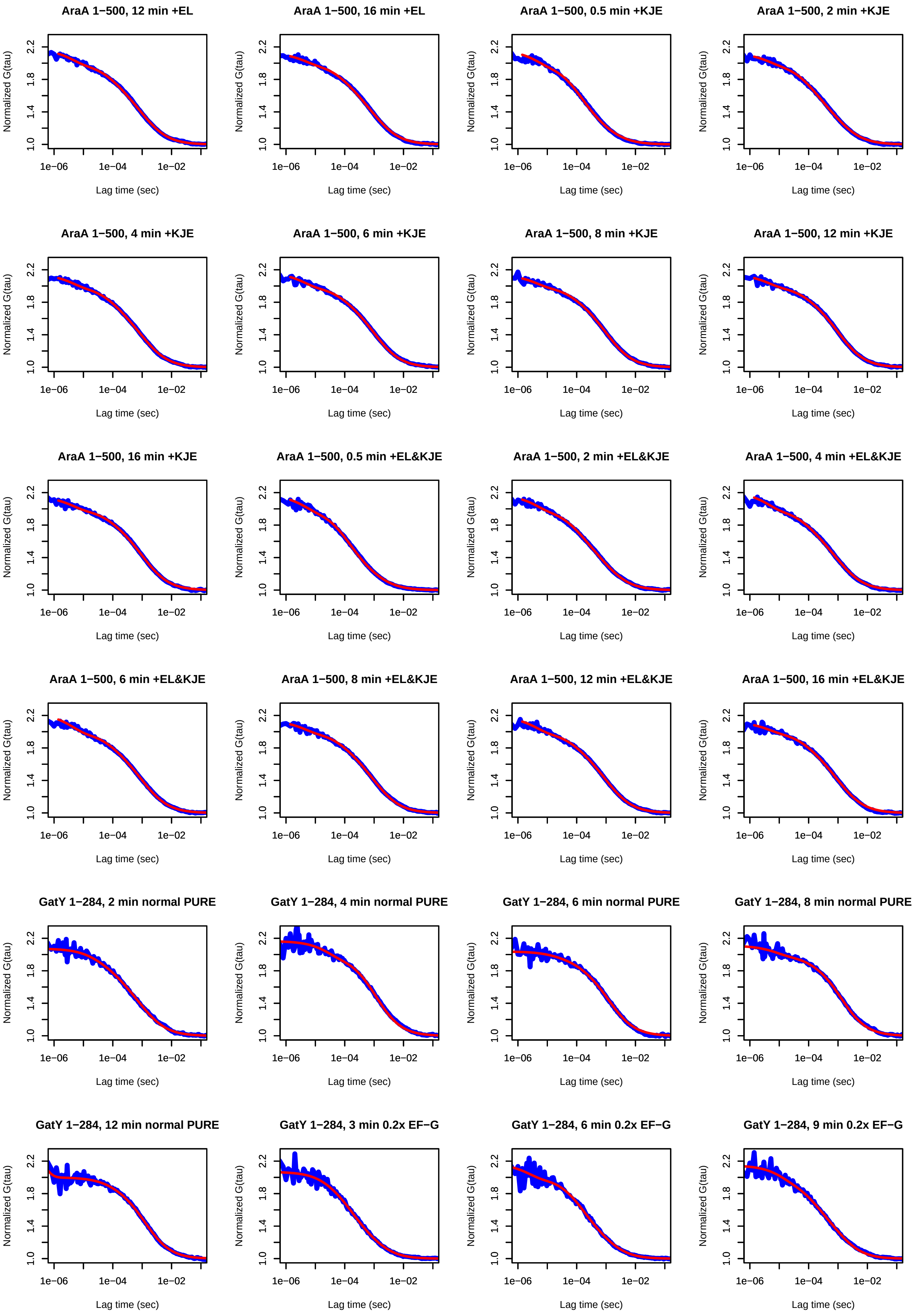
AraA 1−500, 12 min +EL
AraA 1−500, 16 min +EL
AraA 1−500, 0.5 min +KJE
AraA 1−500, 2 min +KJE
AraA 1−500, 4 min +KJE
AraA 1−500, 6 min +KJE
AraA 1−500, 8 min +KJE
AraA 1−500, 12 min +KJE
AraA 1−500, 16 min +KJE
AraA 1−500, 0.5 min +EL&KJE
AraA 1−500, 2 min +EL&KJE
AraA 1−500, 4 min +EL&KJE
AraA 1−500, 6 min +EL&KJE
AraA 1−500, 8 min +EL&KJE
AraA 1−500, 12 min +EL&KJE
AraA 1−500, 16 min +EL&KJE
GatY 1−284, 2 min normal PURE
GatY 1−284, 4 min normal PURE
GatY 1−284, 6 min normal PURE
GatY 1−284, 8 min normal PURE
GatY 1−284, 12 min normal PURE
GatY 1−284, 3 min 0.2x EF−G
GatY 1−284, 6 min 0.2x EF−G
GatY 1−284, 9 min 0.2x EF−G
Normalized G(tau)
1.0
1.4
1.8
2.2
1e−06
1e−04
1e−02
Lag time (sec)

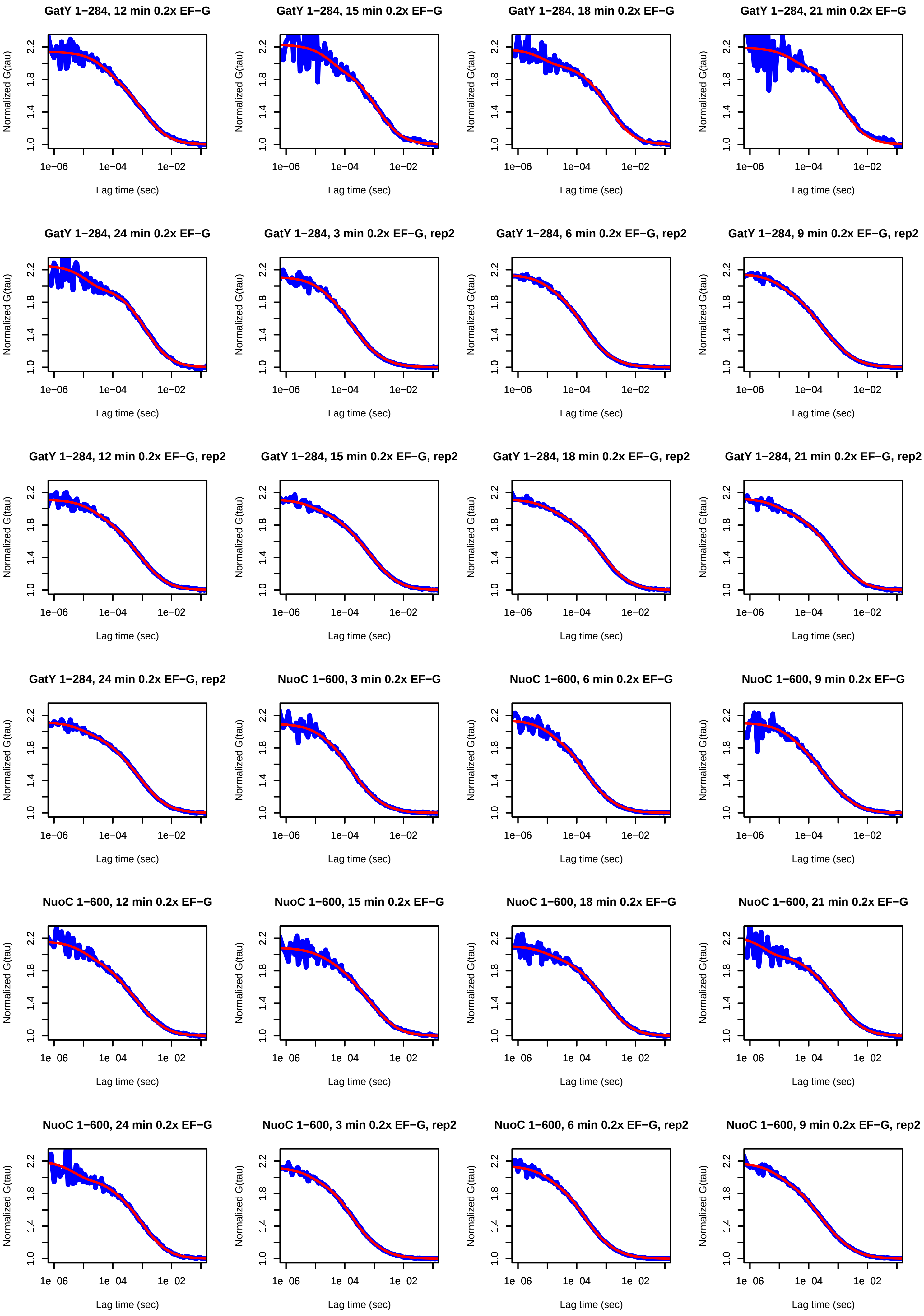
GatY 1−284, 12 min 0.2x EF−G
GatY 1−284, 15 min 0.2x EF−G
GatY 1−284, 18 min 0.2x EF−G
GatY 1−284, 21 min 0.2x EF−G
GatY 1−284, 24 min 0.2x EF−G
GatY 1−284, 3 min 0.2x EF−G, rep2
GatY 1−284, 6 min 0.2x EF−G, rep2
GatY 1−284, 9 min 0.2x EF−G, rep2
GatY 1−284, 12 min 0.2x EF−G, rep2
GatY 1−284, 15 min 0.2x EF−G, rep2
GatY 1−284, 18 min 0.2x EF−G, rep2
GatY 1−284, 21 min 0.2x EF−G, rep2
GatY 1−284, 24 min 0.2x EF−G, rep2
NuoC 1−600, 3 min 0.2x EF−G
NuoC 1−600, 6 min 0.2x EF−G
NuoC 1−600, 9 min 0.2x EF−G
NuoC 1−600, 12 min 0.2x EF−G
NuoC 1−600, 15 min 0.2x EF−G
NuoC 1−600, 18 min 0.2x EF−G
NuoC 1−600, 21 min 0.2x EF−G
NuoC 1−600, 24 min 0.2x EF−G
NuoC 1−600, 3 min 0.2x EF−G, rep2
NuoC 1−600, 6 min 0.2x EF−G, rep2
NuoC 1−600, 9 min 0.2x EF−G, rep2
Normalized G(tau)
1.0
1.4
1.8
2.2
1e−06
1e−04
1e−02
Lag time (sec)

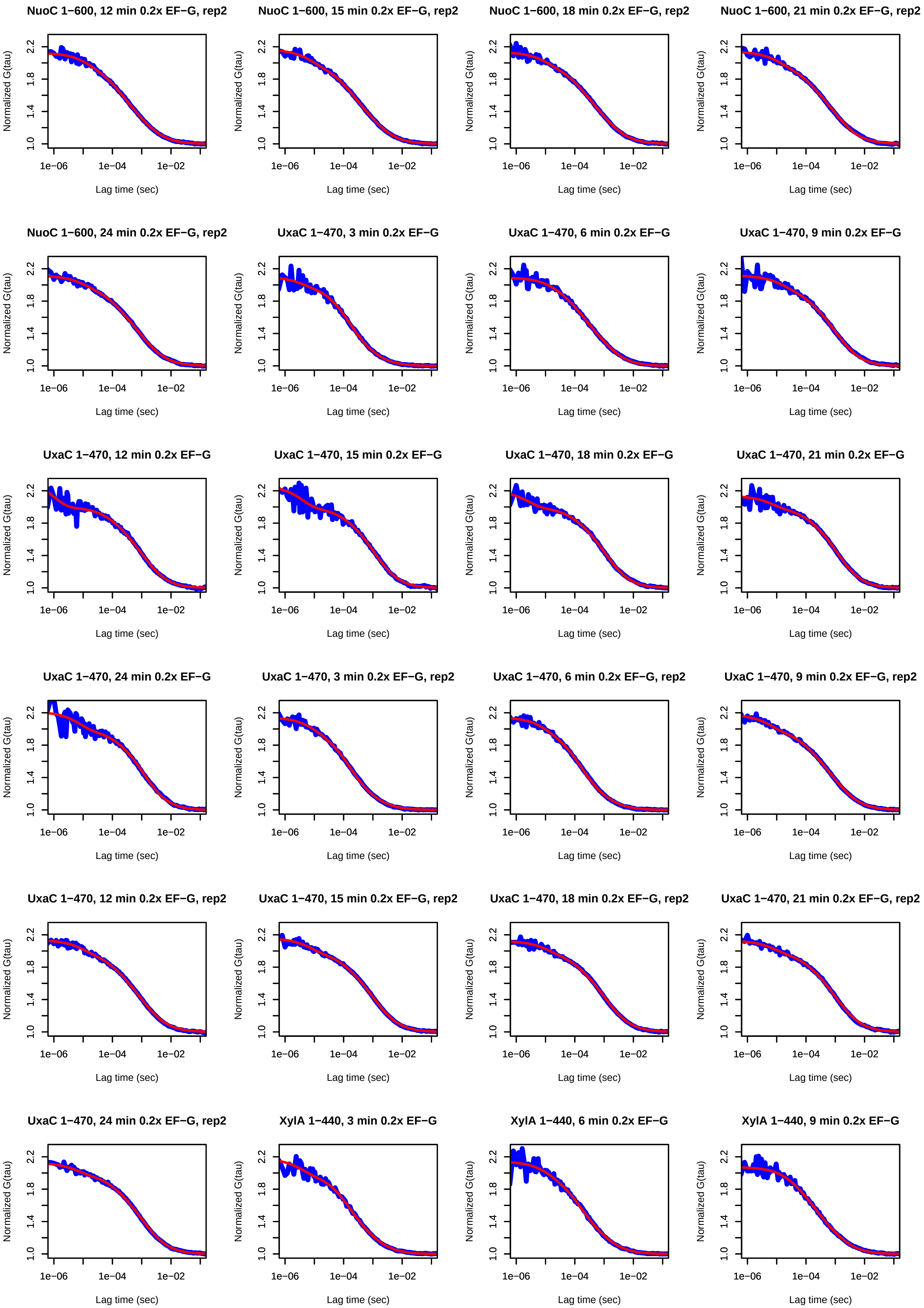

NuoC 1−600, 12 min 0.2x EF−G, rep2
NuoC 1−600, 15 min 0.2x EF−G, rep2
NuoC 1−600, 18 min 0.2x EF−G, rep2
NuoC 1−600, 21 min 0.2x EF−G, rep2
NuoC 1−600, 24 min 0.2x EF−G, rep2
UxaC 1−470, 3 min 0.2x EF−G
UxaC 1−470, 6 min 0.2x EF−G
UxaC 1−470, 9 min 0.2x EF−G
UxaC 1−470, 12 min 0.2x EF−G
UxaC 1−470, 15 min 0.2x EF−G
UxaC 1−470, 18 min 0.2x EF−G
UxaC 1−470, 21 min 0.2x EF−G
UxaC 1−470, 24 min 0.2x EF−G
UxaC 1−470, 3 min 0.2x EF−G, rep2
UxaC 1−470, 6 min 0.2x EF−G, rep2
UxaC 1−470, 9 min 0.2x EF−G, rep2
UxaC 1−470, 12 min 0.2x EF−G, rep2
UxaC 1−470, 15 min 0.2x EF−G, rep2
UxaC 1−470, 18 min 0.2x EF−G, rep2
UxaC 1−470, 21 min 0.2x EF−G, rep2
UxaC 1−470, 24 min 0.2x EF−G, rep2
XylA 1−440, 3 min 0.2x EF−G
XylA 1−440, 6 min 0.2x EF−G
XylA 1−440, 9 min 0.2x EF−G
Normalized G(tau)
1.0
1.4
1.8
2.2
1e−06
1e−04
1e−02
Lag time (sec)

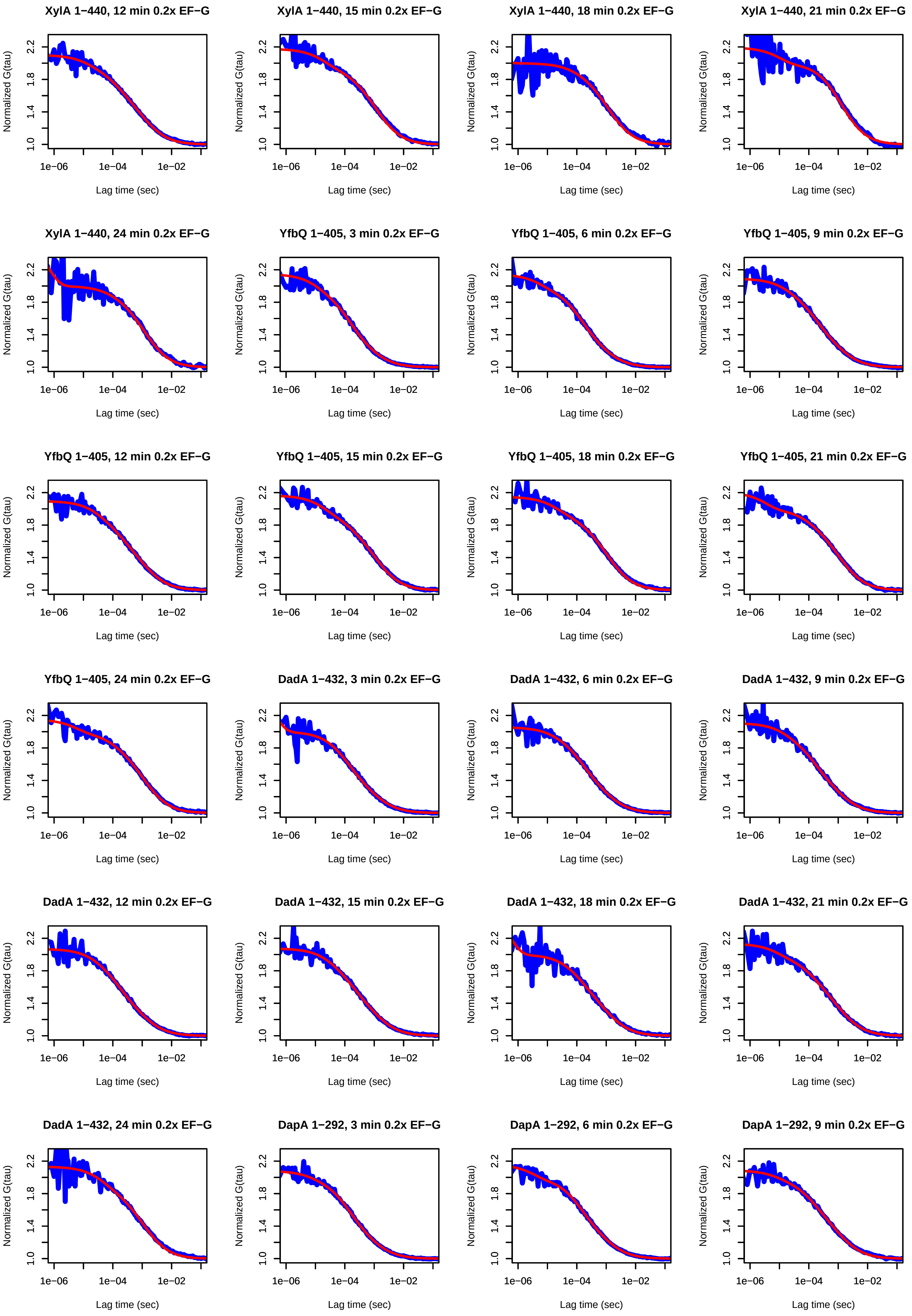
XylA 1−440, 12 min 0.2x EF−G
XylA 1−440, 15 min 0.2x EF−G
XylA 1−440, 18 min 0.2x EF−G
XylA 1−440, 21 min 0.2x EF−G
XylA 1−440, 24 min 0.2x EF−G
YfbQ 1−405, 3 min 0.2x EF−G
YfbQ 1−405, 6 min 0.2x EF−G
YfbQ 1−405, 9 min 0.2x EF−G
YfbQ 1−405, 12 min 0.2x EF−G
YfbQ 1−405, 15 min 0.2x EF−G
YfbQ 1−405, 18 min 0.2x EF−G
YfbQ 1−405, 21 min 0.2x EF−G
YfbQ 1−405, 24 min 0.2x EF−G
DadA 1−432, 3 min 0.2x EF−G
DadA 1−432, 6 min 0.2x EF−G
DadA 1−432, 9 min 0.2x EF−G
DadA 1−432, 12 min 0.2x EF−G
DadA 1−432, 15 min 0.2x EF−G
DadA 1−432, 18 min 0.2x EF−G
DadA 1−432, 21 min 0.2x EF−G
DadA 1−432, 24 min 0.2x EF−G
DapA 1−292, 3 min 0.2x EF−G
DapA 1−292, 6 min 0.2x EF−G
DapA 1−292, 9 min 0.2x EF−G
Normalized G(tau)
1.0
1.4
1.8
2.2
1e−06
1e−04
1e−02
Lag time (sec)

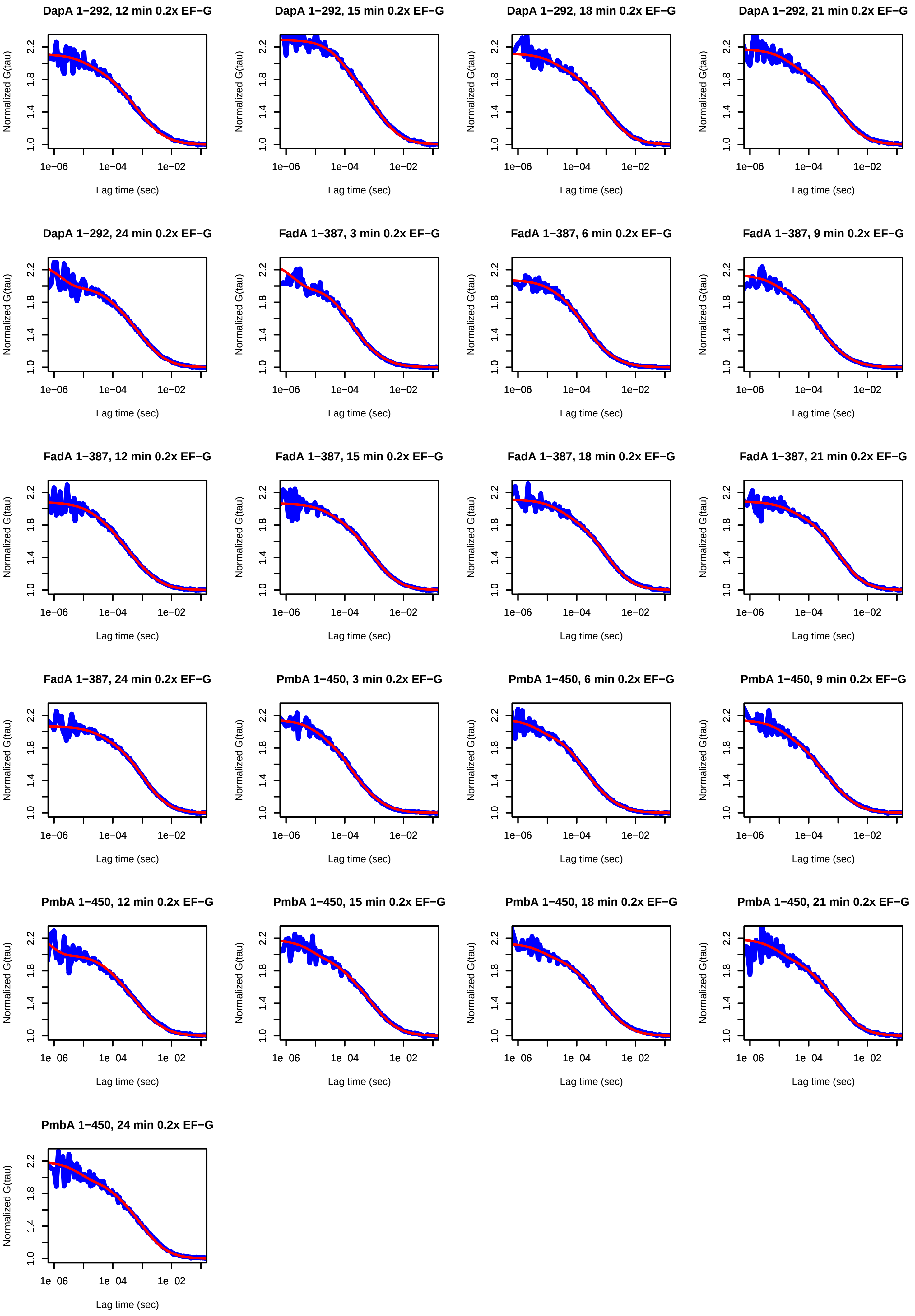
DapA 1−292, 12 min 0.2x EF−G
DapA 1−292, 15 min 0.2x EF−G
DapA 1−292, 18 min 0.2x EF−G
DapA 1−292, 21 min 0.2x EF−G
DapA 1−292, 24 min 0.2x EF−G
FadA 1−387, 3 min 0.2x EF−G
FadA 1−387, 6 min 0.2x EF−G
FadA 1−387, 9 min 0.2x EF−G
FadA 1−387, 12 min 0.2x EF−G
FadA 1−387, 15 min 0.2x EF−G
FadA 1−387, 18 min 0.2x EF−G
FadA 1−387, 21 min 0.2x EF−G
FadA 1−387, 24 min 0.2x EF−G
PmbA 1−450, 3 min 0.2x EF−G
PmbA 1−450, 6 min 0.2x EF−G
PmbA 1−450, 9 min 0.2x EF−G
PmbA 1−450, 12 min 0.2x EF−G
PmbA 1−450, 15 min 0.2x EF−G
PmbA 1−450, 18 min 0.2x EF−G
PmbA 1−450, 21 min 0.2x EF−G
PmbA 1−450, 24 min 0.2x EF−G
Normalized G(tau)
1.0
1.4
1.8
2.2
1e−06
1e−04
1e−02
Lag time (sec)
