# Supplementary material for: Application of fluorescence correlation spectroscopy to investigate the dynamics of a ribosome-associated trigger factor in Escherichia coli
Source: Front Mol Biosci. 2022 Aug 25;9:891128. doi: 10.3389/fmolb.2022.891128 (PMC9452904; doi:10.3389/fmolb.2022.891128)
Supplement: Supplementary file 2 [file DataSheet1.pdf]

Supplementary Material of

Application of fluorescence correlation spectroscopy to investigate the dynamics of a ribosome-associated trigger factor in *Escherichia coli.*

Tatsuya Niwa[1,2], Koki Nakazawa[1], Kensuke Hoshi[1], Hisashi Tadakuma[3], Koichi Ito[4], and Hideki Taguchi[1,2,*]

[1] School of Life Science and Technology, Tokyo Institute of Technology, Yokohama 226-8503, Japan
[2] Cell Biology Center, Institute of Innovative Research, Tokyo Institute of Technology, Yokohama 226-8503, Japan
[3] School of Life Science and Technology & Gene Editing Center, ShanghaiTech University, Shanghai 201210, China
[4] Department of Computational Biology & Medical Sciences, Graduate School of Frontier Sciences, The University of Tokyo, Kashiwa, Chiba 277-8562, Japan

*Correspondence to: taguchi@bio.titech.ac.jp

This document contains the following information:

- Supplementary Figure S1
- Supplementary Figure S2
- Supplementary Figure S3
- Supplementary Table S1
- Supplementary Table S2

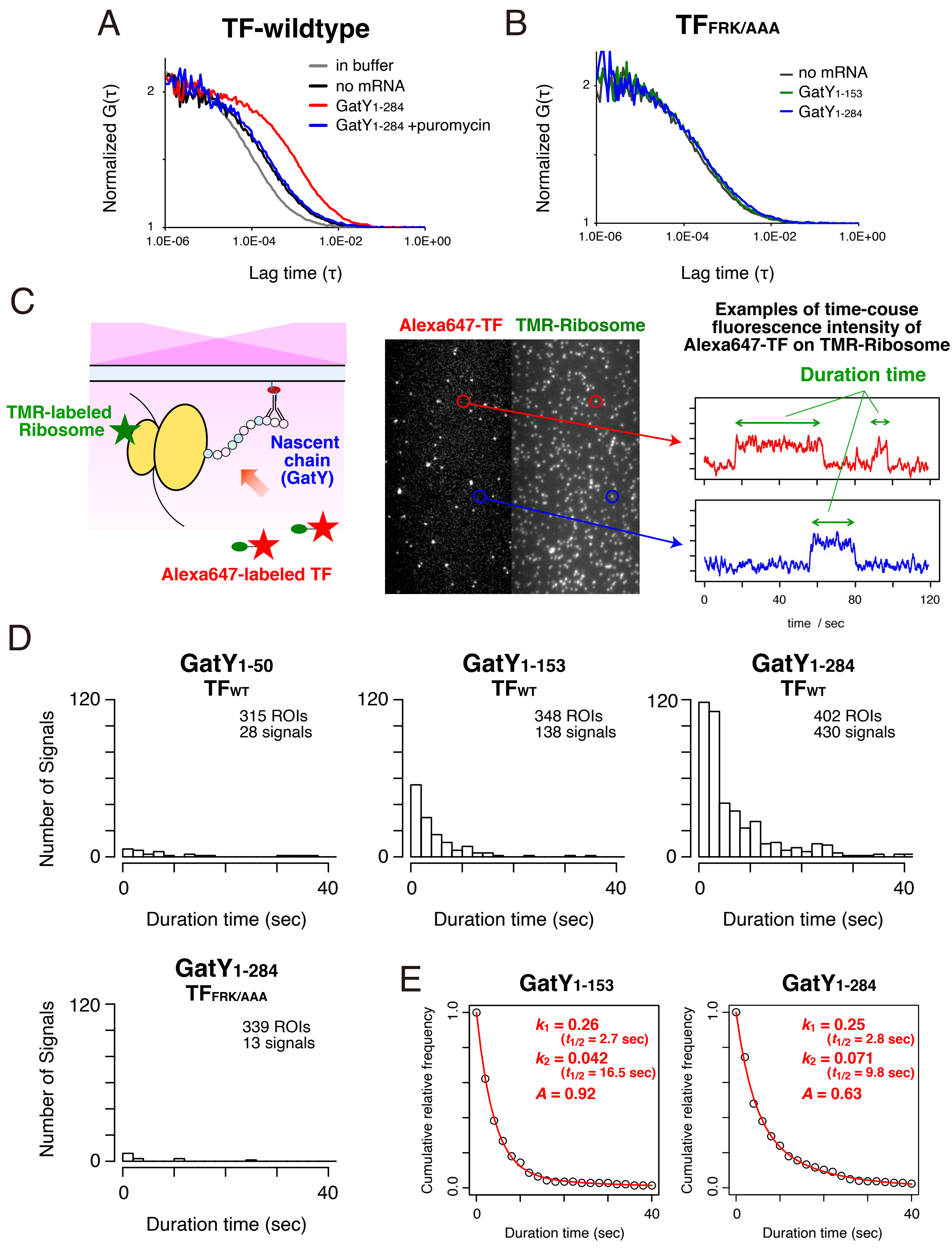

Supplementary Figure S1. FCS measurements and TIRFM observations for investigating interactions between trigger factor (TF) and ribosome-nascent chain complexes (RNCs) *in vitro*.

(A) Autocorrelation curves of Alexa488-labeled TF after the expression of *gatY*$_{1\text{-}284}$ gene and puromycin treatment. The concentration of puromycin was 100 µg/mL (final concentration).

After the addition of puromycin, the sample was incubated at 37 ºC for 5 min before the FCS analysis.

(B) Autocorrelation curves of Alexa488-labeled TF mutant ($TF_{FRK/AAA}$) after the expression of *gatY*$_{1\text{-}153}$ gene or *gatY*$_{1\text{-}284}$ gene.

(C) Schematic illustration (left), snapshot (middle), and examples of the time-course fluorescence intensities (right) of the observations by TIRFM. The ribosome-nascent chain complex was fixed with the biotinylated anti-HA antibody via streptavidin on a PEG-biotin-coated quartz glass slide. The ribosome-nascent chain complex was prepared in a test tube, and the solution was introduced into a slide chamber after the reaction. The fluorescent intensities of Alexa647-labeled TF and TMR-labeled S2-Halo ribosomes were collected at the same time with a splitting filter system. The signal intensity of Alexa647-labeled TF on the TMR-labeled S2-Halo ribosome was acquired for ~2 min, and the duration of the binding of Alexa647-labeled TF on the TMR-ribosome was monitored. As nascent chains, we used GatY truncates ($GatY_{1\text{-}50}$, $GatY_{1\text{-}15}$3, $GatY_{1\text{-}284}$) for the observation.

(D) Histograms of the durations of Alexa647-labeled TF binding at the position of the TMR-ribosome were obtained by the TIRFM observation. Each graph shows the number of regions of interest (ROIs) representing the number of analyzed TMR-ribosome positions, and the number of signals representing the obtained fluorescent signals of Alexa647-labeled TF.

(E) Cumulative relative frequencies of the durations (bins = 2.0 sec) and fitting curves in GatY-153 and GatY-FL. The equation for the fitting is: $y = A \cdot \exp(-k_1 \cdot x) + (1\text{-}A) \cdot \exp(-k_2 \cdot x)$, where the value A indicates the proportion of the component k_1.

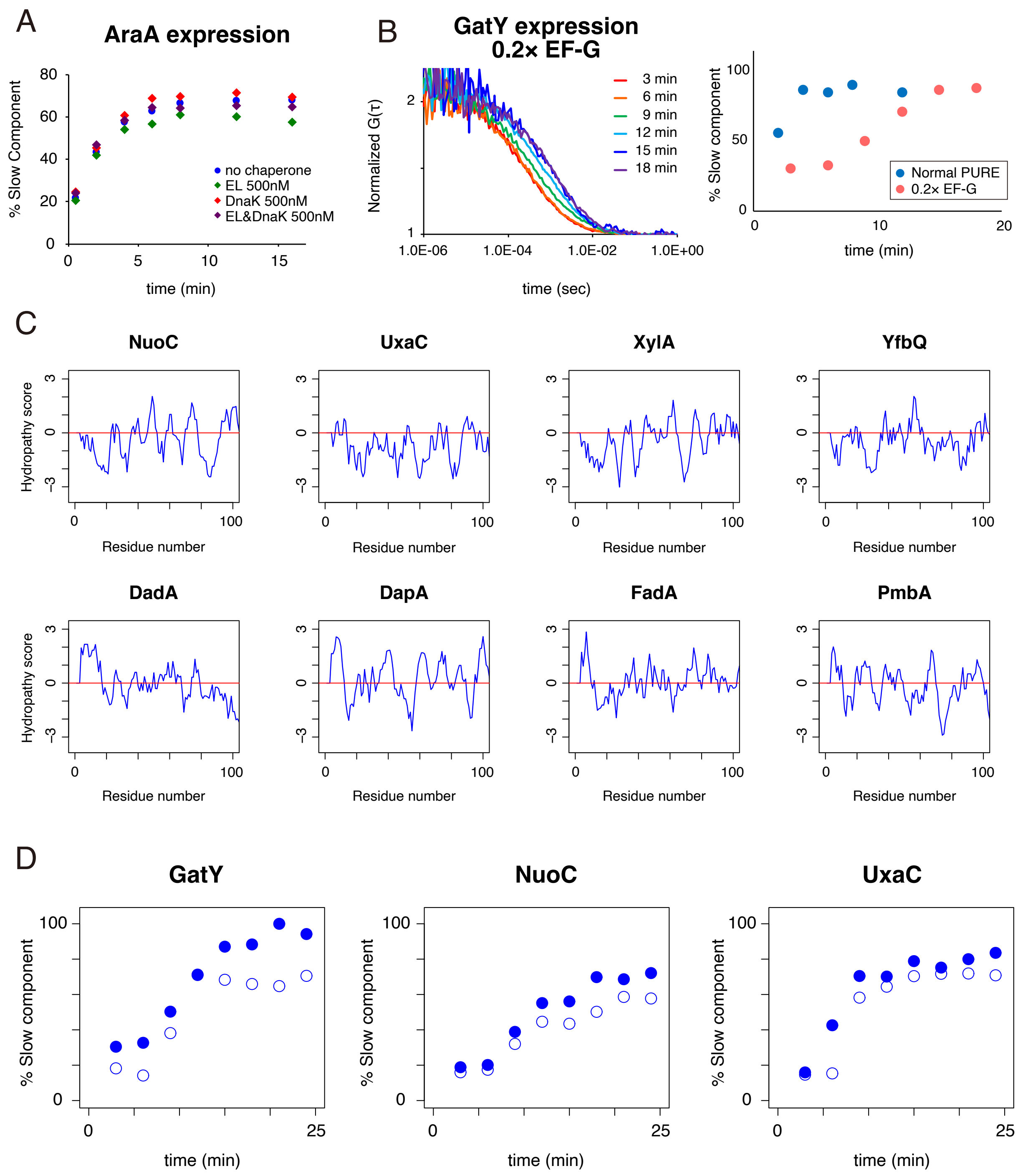

Supplementary Figure S2. Real-time observations of interactions between TF and ribosome-nascent chain complexes by FCS.

(A) Changes in the population of the slow diffusion component with time, using AraA as the model nascent chain instead of GatY. The experimental conditions are the same as those when GatY was used for the observations (Figure 2C).

(B) (Left) Autocorrelation curves at each reaction time of Alexa488-labeled TF during protein synthesis of *gatY*$_{1\text{-}284}$ under the 0.2× EF-G concentration condition. (Right) Time-dependent changes in the populations of the slow diffusion component during the protein synthesis of *gatY*$_{1\text{-}284}$ under the 0.2× EF-G concentration condition. For comparison, the changes under the normal condition (1.0× EF-G) were also plotted.

(C) Hydropathy plots of substrate proteins used in this study. Hydropathy scores for only 100 amino acids from the N termini are shown. The upper four proteins have hydrophilic N-termini and the lower four substrates have hydrophobic N-termini. The window of the hydropathy plot was set as seven.

(D) Reproducibility of the time-dependent changes in the populations of the slow diffusion component during protein synthesis under the 0.2× EF-G concentration condition.

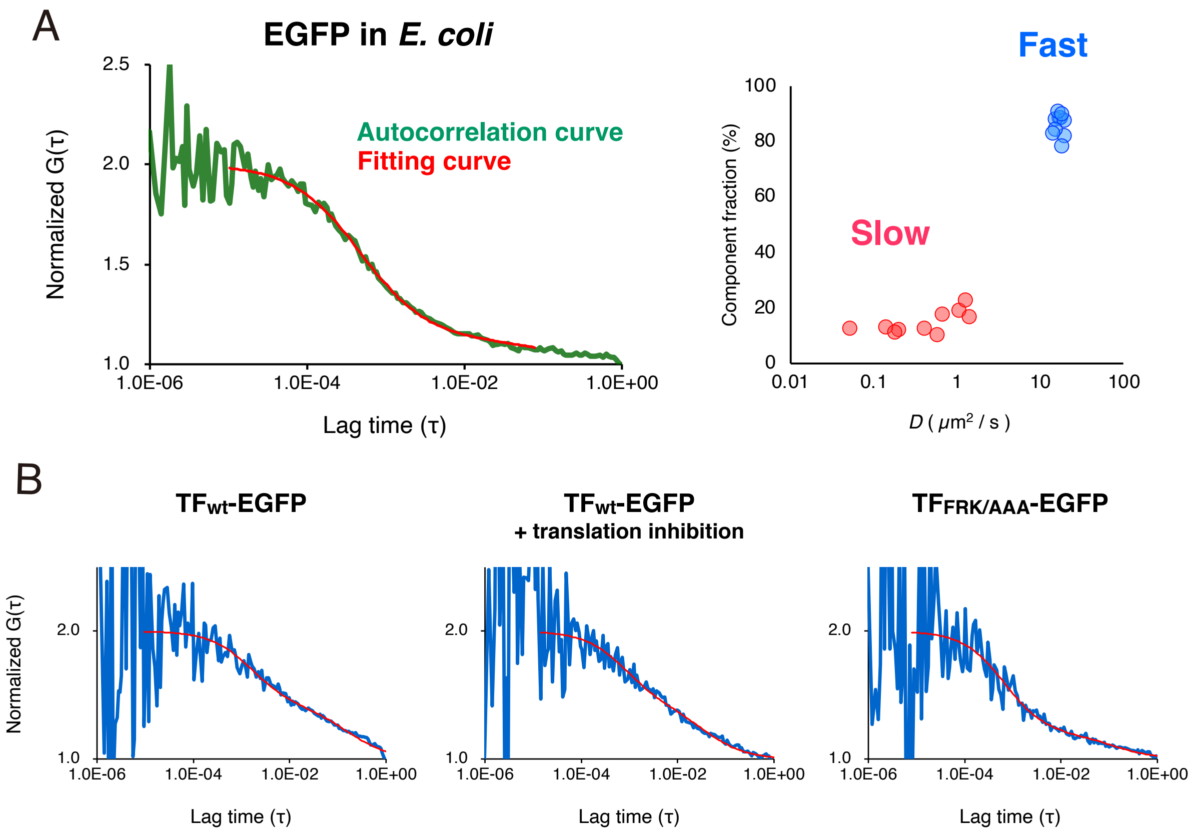

Supplementary Figure S3. ***In vivo*** **observations of the behavior of EGFP-fused TF by FCS.**

(A) Autocorrelation curve of EGFP expressed in *E. coli* cells, and the diffusion parameters of the fast and slow fractions obtained by the two-component fitting. The left panel shows an example of the autocorrelation curve of a single cell expressing EGFP in *E. coli*. The red line indicates the fitting curve with a two-component model. The right panel shows the distribution of the diffusion coefficients and the populations of both the fast and slow components for the investigated cells (N=10).

(B) Examples of autocorrelation curves of single cells expressing EGFP-fused TF in *E. coli*. The red line indicates the fitting curve with a two-component model.

Supplementary Table S1 List of the plasmids for the templates to express with the PURE system used for FCS and TIRFM observations.

No.	Name	Information	Source
1	pET-HA-GatY	For Figure1 and Supplementary Figure S1.<br><br>The sequences of the primers for truncated products were listed in Supplementary Table S2 (No. 1-4).	This study
2	pTac-GatY	For Figure 2 and Supplementary Figure S2.<br><br>The T7 promoter sequence was added by PCR reaction. The sequences of the primers were listed in Supplementary Table S2 (No. 5-14).	Fujiwara *et al., EMBO J.* 2010
3	pTac-AraA		
4	pTac-NuoC		
5	pTac-UxaC		
6	pTac-XylA		
7	pTac-YfbQ		
8	pTac-DadA		
9	pTac-DapA		
10	pTac-FadA		
11	pTac-PmbA		

Supplementary Table S2 Primer sequences for amplifying truncated DNA fragments.

No.	Position	Direction	Sequence
1	T7 promoter	5′-3′	CGCGAAATTAATACGACTCACTATAGGG
2	*gatY* $_{1-50}$	3′-5′	CGTTCCGGCGATGATGACCG
3	*gatY* $_{1-153}$	3′-5′	CAACGCATCGGCTTCATTGAC
4	*gatY* $_{1-284}$	3′-5′	TGCCCTGCCCTCGCAG
5	Addition of T7 promoter sequence for pTac plasmid	5′-3′	GAAATTAATACGACTCACTATAGGGAGACCACA ACGGTTTCCCTCTAGAAATAATTTTGTTTACTGC AGGTAAGGAGATATACAT
6	*araA* $_{1-500}$	3′-5′	GCGACGAAACCCGTAATACACTTC
7	*nuoC* $_{1-600}$	3′-5′	TGCCCTGCCCTCGCAG
8	*uxaC* $_{1-470}$	3′-5′	GCGACGAAACCCGTAATACACTTC
9	*xylA* $_{1-440}$	3′-5′	GCGGTCCACATCTGACATAACAAAATC
10	*yfbQ* $_{1-405}$	3′-5′	GTTCAGTTCAATGGCGAAGTAGTC
11	*dadA* $_{1-432}$	3′-5′	TTTGTCGAACAGATAATGGTTTACCAG
12	*dapA* $_{1-292}$	3′-5′	CAGCTGATGATAACCAGAAAGGAAAC
13	*fadA* $_{1-387}$	3′-5′	GCTGTGTGCGCCATGTAAATG
14	*pmbA* $_{1-450}$	3′-5′	CAGCAAACCGGCATGCTTAAG
